# Combination of the EP and Anti-PD-1 Pathway or Anti-CTLA-4 for the Phase III Trial of Small-Cell Lung Cancer: A Meta-Analysis

**DOI:** 10.1155/2021/6662344

**Published:** 2021-05-22

**Authors:** Abdulhakim A. A. Abunasser, Jinmin Xue, Ehab J. A. Balawi, Yuxi Zhu

**Affiliations:** ^1^Department of Oncology, The First Affiliated Hospital of Chongqing Medical University, Chongqing City 400016, China; ^2^Department of Oncology, Jinshan Hospital of the First Affiliated Hospital of Chongqing Medical University, Chongqing City 400016, China; ^3^Chongqing Clinical Cancer Research Center, Chongqing City 400016, China; ^4^Department of Neurosurgery, Spine Surgery Department, The First Affiliated Hospital of Chongqing Medical University, Chongqing City 400016, China; ^5^Department of Oncology, The First Affiliated Hospital of Chongqing Medical University, No. 1 Youyi Road, Yuzhong District, Chongqing City 400016, China

## Abstract

The morbidity and mortality of lung cancer remain one of the highest among multiple cancers, respectively. Small-Cell Lung Cancer (SCLC) accounts for around 10%–15% of all lung cancers. Approximately two-thirds of the diagnosed SCLCs are in extensive stage (ES). Decades later, we still rely on the same traditional regimen with etoposide and platinum (EP) as the mainstay of treatment with poor prognosis. This meta-analysis aims to assess the effect of adding Immune Checkpoint Inhibitors (CPIs) such as (ipilimumab, atezolizumab, pembrolizumab, and durvalumab) to the traditional EP regimen for small-cell lung cancer extensive stage (ES-SCLC). We searched through PubMed looking for studies that compare between EP and CPIs, with EP alone, and only Phase III randomized controlled trials were considered eligible for this study. A total of 3645 papers were the results of the initial search, and only 4 studies met our criteria. Each investigator extracted the data independently using the PRISMA MODEL (Preferred Reporting Items for Systematic Reviews and Meta-Analyses) guideline. Each author used a prespecified sheet. The primary endpoint was to calculate OS (overall survival) and PFS (progression-free survival) hazard ratios for both arms. We found that adding EP plus CPIs increased both OS (HR, 95% CI 0.80 [0.70, 0.93], *P* = 0.0001, *I*^2^ = 49%) and PFS (HR95% CI 0.81 [0.74, 0.88], *P* < 0.00001, *I*^2^ = 0%). On the other hand, ORR (overall response rate) was not affected by the addition of CPIs to EP compared to EP alone, and the same was true for adverse events. To conclude, CTLA-4 alone is not encouraging, but PD-1/PD-L1 adds survival benefits. A combined treatment regimen shows to be more effective, improving overall survival rate Durvalumab and atezolizumab showed improvement for OS, but pembrolizumab and ipilimumab did not show a significant increase of OS over EP; however, pembrolizumab showed significant prolongation of the disease-free period.

## 1. Introduction

Lung cancer is the most diagnosed cancer and the most common cause of death among all types of cancers. Based on histological subtypes, it mainly consists of two categories: Small-Cell Lung Cancer (SCLC), which accounts for almost 10%–15% and Non-Small-Cell Lung Cancers (NSCLCs), which account for 85% of all lung cancers [1].

Small-Cell Lung Cancer (SCLC) is one of the most aggressive lung cancers [2]. Depending on the existence of metastasis, SCLC is classified as either extensive-stage SCLC or limited-stage-“disease” small-cell lung cancer. For newly diagnosed patients, the initial aim is to determine the distant metastasis [3]. SCLC is strongly associated with smoking [3]. In recent years, the incidence ratio among male and female has become almost equal (1 : 1), as smoking has become more common among women. One-third of SCLC cases present with limited-stage SCLC, and almost two-thirds of the diagnosed SCLC cases present with extensive disease. LS-SCLC could be curable, while for most ES-SCLC cases, treatment is not feasible. Surgical treatment is indicated only at early stage of LS-SCLC, and only 5% or less of treated cases could benefit from surgical resection and adjuvant chemotherapy “stage I (T1-2 N0)” [4]. The standard for LS-SCLC is 4/6 cycle of cisplatin or carboplatin and etoposide with concurrent radiotherapy, in addition to prophylactic whole brain radiation for the patient with good ECOG performance score. This usually results in an objective response rate above 90% [5]. ES-SCLC platinum-based chemotherapy is indicated only for palliative treatment. Chemotherapy achieves an objective response in 60% to 70%. Either cisplatin or carboplatin can be used in the EP regimen as both show the same effectiveness, but carboplatin is less toxic [5]. Additionally, if the patient has good performance status, we can use radiotherapy. The CREST study has shown significant improvements of 2-year OS with radiotherapy for patients with ES-SCLC [6]. As for patients with SCLC, 59% to 69% of them will eventually develop brain metastasis. Prophylactic cranial irradiation has proved to have 5.4% absolute survival advantage. Although chemotherapy improves the quality of life for most patients, relapse is mostly invincible for ES-SCLC and only 10% of patients survive for two years [6, 7].

Three types of CPIs are currently used and approved, CTLA-4 inhibitors which include ipilimumab, PD-1 inhibitors (pembrolizumab and nivolumab), and PD-L1 inhibitors (atezolizumab and durvalumab). CTLA-4 is the beginning point of activation for inactive T-lymphocyte and a mediator of the suppressor activity of regulatory T-lymphocytes [8]. Inhibition or a deficiency in CTLA-4 could initiate a general immune response at many organs. The PD-1 role is to protect the organs from exaggerated immune responses and autoimmunity [9]. Unlike CTLA-4, PD-1 inhibitors regulate only peripheral activated T-lymphocyte. Additionally, it is usually expressed at every activated lymphocyte including NK lymphocyte. The PD-1 ligands known as PD-L1 and PD-L2 are expressed in a large variety of cells including hematopoietic cells, pancreatic islets, and cancer cells. Interferon is the main inducer for PD-L overexpression at cells [9].

With the advent of CPIs, the argument for possible implications for SCLC, especially for the ED-SCLC, has become more convincing [10]. In 2016, the cytotoxic T-lymphocyte-associated protein 4 (CTLA-4) antibody (ipilimumab) combined with chemotherapy tested on SCLC showed no significant effects [11]. On the other hand, other immunotherapies, such as pembrolizumab, appeared to be effective when combined with chemo in treating NSCLC [12, 13]. In 2018, the first anti-programmed-death-ligand 1 receptor (PD-L1) atezolizumab combined with platinum-based chemotherapy had been studied on SCLC. It was the first drug combination to demonstrate significant differences in survival rate over EP [14]. Encouragingly, afterwards, the CASPIAN trial showed substantial results, reporting some progress in SCLC [15]. According to the most recent study on pembrolizumab, it has also been proven effective for SCLC [16]. Interestingly, although both anti-CTLA-4 and anti-PD-1 pathways are CPIs, their efficacy in regard to SCLC is different. Four drugs have been approved, two PD-1 inhibitors (pembrolizumab, nivolumab) have been approved as third-line treatment in ES-SCLC, and two PD-L1 inhibitors (atezolizumab and durvalumab) have been approved in first-line treatment ES-SCLC [17].

Therefore, this study aims to summarize the data from these clinical trials to estimate the efficacy and toxicity of the anti-PD-1 pathway or anti-CTLA-4 plus EP for ED-SCLC.

## 2. Methods

This review followed the checklist of the Preferred Reporting Items for Systematic Reviews and Meta-Analyses (PRISMA).

### 2.1. Study Eligibility and Identification

PubMed was searched using the following keywords: Nivolumab, Ipilimumab, Atezolizumab, Pembrolizumab, Checkmate, impower, small-cell lung cancer, keynote, and lung cancer. Studies were restricted to English language published or presented up to July 15, 2020. The Phase III randomized controlled trials comparing CPIs plus EP versus EP alone of SCLC were only considered eligible.

### 2.2. Data Extraction

From the four qualified studies for the meta-analysis, the data have been extracted by the authors and separate independent reviewers to ensure accuracy. We extracted the following items for each included trial: authors, study design, year of publication, place of publication, age, gender, smoking status, Eastern Cooperative Oncology Group (ECOG) Performance Status, PD-L1 level, lung cancer subtypes, metastasis, drugs, the hazard ratio of OS, PFS, ORR, and adverse events (AEs) for both arms, any other clinical outcomes, and the follow-up period. Disagreements were resolved by discussion. The data have been gathered according to the prespecified sheet. We summarized primary and subgroup endpoints in addition to the general characteristics included in each RCT. Each author collected the data separately. Data were revised by the authors and the supervisor.

### 2.3. Statistical Analysis

The primary objective was to investigate treatment effects in both arms by analyzing the pooled overall survival rate, the subgroup's OS, and the pooled PFS in patients with advanced SCLC.

The secondary outcomes included the pooled risk of adverse events and ORR. We used the Cochrane's Q statistic to assess between-study heterogeneity and calculated the I^2^ statistic, which estimates the percentage of total variation across studies due to heterogeneity rather than chance.

The pooled estimates of OS and PFS outcomes were represented by hazard ratios (HRs) with corresponding 95% confidence intervals (CIs) and *P* values using the inverse-variance-weighted method.

ORR was calculated by using the odds ratio, for a frequency of adverse events RR, 95% CIs, and *P* values using the Mantel–Haenszel method. We used the *I*^2^ Cochran Q test to calculate heterogeneity across the trials and between the two arms. If the *I*^2^ was higher than 50%, the random effect was applied. A funnel plot of the effect size of each study was used to assess the publication bias.

Sensitivity analysis was performed through the exclusion of trials one by one in each comparison. Review manager 5.3 was used to apply the analysis. The Martin Reck 2016 study with anti-CTLA-4 is the only study that shows some weight effect on the result, but only for subgroups without a major effect on the pooled results or outcome or conclusion.

## 3. Results

### 3.1. Data Search and Study Description

A total of 3645 papers were the results of the initial search. ‏Two hundred and thirty papers were excluded because of duplication, and 3109 were excluded after title screening. Two hundred and ninety-one papers were covered by the final assessment. After reading the abstract, four studies remained for full reading. After full-text reading, these four studies were included (supplementary figure 1).

Quality assessment was performed, and six aspects were tested including (1) randomization process (selection bias), (2) deviations from intended interventions, (3) measurement of the outcome, (4) missing outcome data (attrition bias), (5) selection of the reported result (reporting bias), (6) and overall bias (supplementary figure 2). According to the Cochrane collaboration's tool, the RCT was considered low risk of bias if all domains were at low risk of bias, unclear risk of bias if there was an unclear risk of bias of at least one domain, and high risk of bias if at least one domain was scored as being at a high risk of bias. The quality assessment agreement between reviewers was evaluated via *Cohen's kappa coefficient*, where the *kappa* value was 0.8.

The main characteristics of the included trials are presented in Table 1. The patient characteristics were well balanced for most studies, as supplementary table 1 shows. The overall patient number for CPIs plus EP is 1263 and EP is 1262.

### 3.2. Primary End-Point Analysis

**Figure 1:**
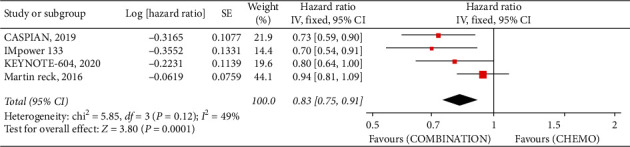
The overall survival hazard ratio between the combination with ICIs and EP alone.

The primary point of analysis shows that combined EP with all kinds of CPIs (*anti-PD-1 pathway or anti-CTLA-4*) indicated survival advantages over EP alone, as displayed down below. Pooled OS (HR 95% CI 0.80 [0.70, 0.93], *P* = 0.0001, *I*^2^ = 49%) showed that combined therapy significantly improved the OS more than that of EP alone Figure 1.

**Figure 2:**
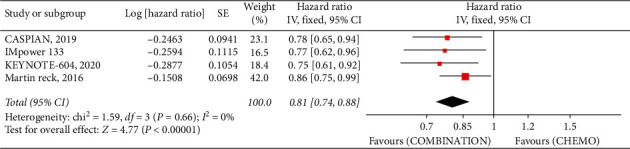
Progression-free survival hazard ratio between combination with ICIs and EP alone.

Pooled PFS was also significantly higher in combined therapy (HR95%, CI 0.81 [0.74, 0.88], *P* < 0.00001, *I*^2^ = 0%), than EP alone (Figure 2), and the same for OS, even with ipilimumab.

**Figure 3:**
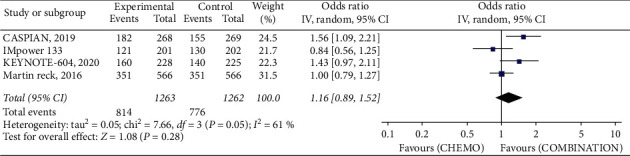
The overall response rate odds ratio with and without ICIs.

ORR odds ratio (IV, random, 95% CI 1.16 [0.89, 1.52], *P* = 0.05, *I*^2^ = 61%) of combined therapy showed no significant advantage over EP alone (Figure 3) with only 16 patients of the combination arm experiencing complete response and 6 from EP arm.

### 3.3. Safety Analysis

**Figure 4:**
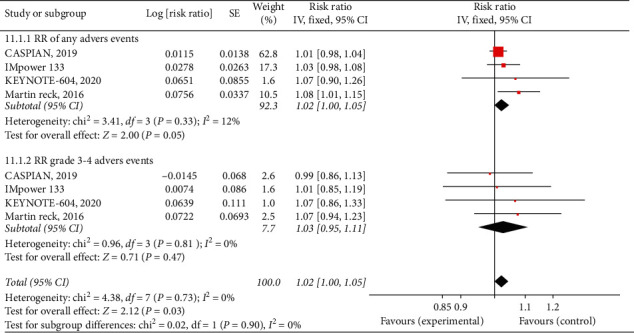
Risk ratio difference for any adverse events and for grade 3-4 adverse events between two groups.

The safety analysis was performed only for patients who at least received one dose of the intended treatment. We analyzed the RR, for any adverse events risk ratio (IV, fixed, 95% CI 1.02 [1.00, 1.05], *P* = 0.05, *I*^2^ = 12%) and for 3-4-grade adverse events risk ratio (IV, fixed, 95% CI 1.03 [0.95, 1.11], *P* = 0.47, *I*^2^ = 0%). According to our analysis, a significant increase was not demonstrated by adding CPI to EP for extensive-stage SCLC Figure 4.

### 3.4. Subgroup Analysis

The analysis of OS of subgroups showed that the benefits of adding CPIs to EP were almost universal and the only exception was the preexistence of brain or liver metastasis; otherwise, all subgroups showed almost equal OS (HR [95% CI]).

The OS in case of brain metastasis, the hazard ratio was 1.19 (IV, random, 95% CI, [0.83, 1.71]), supplementary figure 8, and for liver metastasis, the hazard ratio was 0.80 (IV, random, 95% [0.65, 0.99]), supplementary figure 9, showing no significant advantage over EP therapy alone.

The PFS in case of liver metastasis (HR 95%, CI 0.86 [0.68, 1.07], *P* = 0.17, *I*^2^ = 0%), supplementary figure 6, and brain metastasis (HR 95% CI 1.03 [0.66, 1.61], *P* = 0.89, *I*^2^ = 0%) showed no statistically significant difference by the combination of CPIs and EP (supplementary figure 7).

As for ECOG status, the hazard ratio of ECOG0 was 0.94 (IV, fixed, 95% CI 0.94 [0.78, 1.13], *P* = 0.39, *I*^2^ = 69%), and the sensitivity test showed that the Martin Reck 2016 study had high weight over the result. After we omitting it, the hazard ratio was 0.72 (IV, fixed, 95% CI 0.72 [0.56, 0.92], *P* = 0.010, I2 = 0%) (supplementary figure 3).

As for ECOG1, the hazard ratio was 0.86 (IV fixed, 95% CI 0.86 [0.77, 0.97], *P* = 0.01, *I*^2^ = 46%).

As for AGE <65, the hazard ratio was 0.90 (IV, random, 95% CI 0.90 [0.75, 1.09], *P* = 0.13, *I*^2^ = 47%), and when we omitted the Martin Reck 2016 study, the hazard ratio was 0.81 (IV, fixed, 95% CI 0.81 [0.68, 0.97]).

In case of AGE ≥ 65, the hazard ratio was 0.79 (IV, random, 95% CI 0.79 [0.58, 1.06], *P* = 0.11, *I*^2^ = 73%), supplementary figure 5, and when we omitted the Martin Reck 2016 study, the hazard ratio was 0.70 (IV, fixed, 95% CI 0.70 [0.58, 0.85])

The hazard ratio for male was 0.84 (IV, random, 95% CI 0.84 [0.69, 1.03], *P* = 0.09, *I*^2^ = 63%) and 0.82 for female (IV, random, 95% CI 0.82 [0.64, 1.06], *P* = 0.14, *I*^2^ = 48%) (supplementary figure 4). The sensitivity test showed that the Martin Reck 2016 study had high weight over the result, and after we omitted it, the hazard ratio was 0.72 (IV, fixed, 95% CI 0.76 [0.65, 0.88]) and 0.73 (IV, fixed, 95% CI 0.73 [0.58, 0.93]), respectively.

## 4. Discussion

The majority of meta-analyses are about the impact of CPIs on NSCLC, and those that address SCLC are mostly general reviews. There is one meta-analysis about the effect of PD-1/PD-L1 inhibitors on SCLC. To our knowledge, this is the first meta-analysis to compare the effects of all CPIs.

CPIs have three types, anti-CTLA-4, anti-PD-1, and anti-PD-L1 Theoretically, CTLA-4 is responsible for the initiation of immune suppression reaction, and it was estimated that anti-CTLA-4 might be the most critical of all CPIs in immunotherapy. However, the clinical data from these studies show that PD-L1/PD-1 inhibitors, namely, durvalumab, pembrolizumab, and atezolizumab, are more effective than the CTLA-4 inhibitor, namely, ipilimumab [18].

This meta-analysis, based on the published data in the eligible four phase III trials, showed that CPIs combined with EP in ED-SCLC achieved significantly higher OS (HR 95% CI 0.80 [0.70, 0.93], *P* = 0.0001, *I*^2^ = 49%). CPIs plus EP also improves PFS (HR95%, CI 0.81 [0.74, 0.88], *P* < 0.00001, *I*^2^ = 0%) without the significant increase of AEs (RR IV, fixed, 95% CI 1.02 [1.00, 1.05], *P* = 0.05, *I*^2^ = 12%). The median overall survival rate increased in the range 0 to 4 months over the conventional EP regimen; the range also improved median progression-free rate from 0 to 1 months.

For the first time, a combined treatment regimen shows to be more effective, improving the overall survival rate [19]. Durvalumab and atezolizumab showed improvement for OS, but pembrolizumab and ipilimumab did not show significant increase of OS over EP, but pembrolizumab showed significant prolongation of the PFS [16].

As CPIs have broad activity and their effectiveness ranges from 15%–90% depending on cancer type, [20] there is no need for high PD-L1 level to ensure efficacy. Furthermore, addition of these drugs increases the long-lasting effectiveness of chemotherapy [20]. Only keynote-604 (pembrolizumab) considered the PD-L1 level, which had no significant rules for pembrolizumab in SCLC [21]. This finding is consistent with some studies on other types of cancer [22]. IMpower133 showed no evidence of association of tumor mutational burden with outcomes. It is well established that chemotherapy has a synergistic effect on CPIs. Correspondingly, recent studies have shown that chemotherapy upregulates PD-L1 expression in tumor cells [23, 24]. Additionally, CPIs contradict the immunosuppression effect of chemotherapy and promote the immune system to eliminate tumor cells [10, 12]. Although CPIs improved the OS and PFS, there is no difference in ORR between the two groups. CPIs could not function until they activate cancer infiltrate lymph (CIL) cells. On the other hand, SCLC is sensitive to the chemotherapy itself in the first-line treatment; therefore, it is difficult for CPIs plus chemotherapy to show its benefits over chemotherapy alone in response rate. Checkmate 32 demonstrated that using nivolumab and ipilimumab together could improve the ORR; however, it increases the toxicity, which could decrease the tolerance and the duration of response [25]. Nevertheless, CPIs improved the duration of response of SCLC, especially when one drug was used, even as a third-line drug [15, 25]. The most effective CPIs in ED-SCLC are PD-1/PD-L1 inhibitors.

According to the available data in the literature, there is no reliable marker for CPIs effectiveness. Luterstein et al. suggest that in NSCLC, previous radiotherapy could indicate a prognostic factor for how the patients will respond to CPIs, but there has been no such research for SCLC [26]. Neither study used radiotherapy as part of the treatment regimen, only prophylactic whole brain radiotherapy but not chest.

The CPIs plus EP regimen is generally well tolerated. The drop rate was almost similar between the two groups whether because of withdrawal, physician decision or because of adverse events across the studies, or discontinuation of the treatment because of AEs. In Reck et al.'s study, there were 85 patients for the combined group vs. 9 patients for EP [11], at CASPIAN, 35 vs. 18 [15], and at the KEYNOTE-604 study, 31 vs. 13 [16]. For most cases, only the expected increase in immune-related adverse events was observed, but it was mostly tolerable.

Regarding the patients' physical and general situation, it was reported using the Eastern Cooperative Oncology Group (ECOG) scale that ECOG1 performance status records reported at the beginning of the treatment had additional advantages of adding CPI [13]. In contrast to several previous studies that reported a disparity between male and female in response to CPIs, our study showed no significant effect of gender on the response to CPIs. However, these studies' findings are inconsistent as some have shown advantages for male and some for female. It should be put in consideration that our study sample is smaller than the study sample of those studies [22].

At the present, six cycles of PE in addition to prophylactic whole brain radiation is the standard treatment for ES-SCLC [19]. For years, no other drugs have been approved as standard for ED-SCLC [27]. Genetically, SCLC is more complicated than non-small-cell lung cancer. SCLC does not have the mutation that is usually used by target therapy such as epidermal growth factor receptor (EGFR) [28]. Instead, it has more point mutations in genes such as P53 and BCL, which make it resistant for traditional treatment [29, 30]. Hence, CPIs are something to look forward to [27].

All four studies included patients with brain metastasis. The brain and liver metastasis are worth mentioning at the beginning of treatment because of their correlation with poor prognosis [31]. The microenvironment of the brain and liver might not be suitable for CPIs in SCLC. Moreover, most of the patients with brain metastasis need dexamethasone treatment. There is no advantage of CPIs for PFS and OS in the existence of brain and liver metastasis [31, 32].

The limitation of this study is that it only included 4 RCTs, which could be considered a small number. Nevertheless, all of them are phase III studies and of good quality.

## 5. Conclusions

This analysis shows that CPIs are the first drugs to show significant and sustained improvements in overall survival for patients with ES-SCLC. These drugs have proven to be well tolerated and correlated with a minimal increase in AEs [33]. These findings further support the idea of adding this form of drugs to first-line treatments of ED-SCLC. Although we are still so far from totally replacing EP even as second-line treatment as in checkmate 331 [34], the findings suggest substantial undeniable additional benefits from adding CPIs. This study shows that different CPIs have different outcomes. Although some drugs are approved for clinical use in SCLC, this study shows that a lot of clinical trials are needed on this topic to study more options, for example, combining different kinds of CPIs. Generally, now we are at a better position than before in regard to SCLC treatment.

## Figures and Tables

**Table 1:** The included studies, patient's number of each study, and the name of drugs that included in each one. Moreover you can see the median overall survival and PFS (ITT: intention-to-treat population). For full patient characteristics, see supplementary table 1.

Study ID	PT no.	Experimental		CHEMO				OS COMB	OS CHEM	PFS COMB	PFS CHEMO
		ITT	Safety	ITT^*∗*^	Safety	Experimental drug name	Cancer types	Median months		Median months	
Martin reck 2016 ipi + etoposide and platinum small cell	1132.00	566.00	562.00	566.00	561.00	Ipilimumab + EP	SCLC	11	10.9	4.6	4.4
Impower 133 2018 small cell	403.00	201.00	198.00	202.00	196.00	Atezolizumab + EP	SCLC	12.3	10.3	5.2	4.3
CASPIAN 2019 small cell	683.00	268.00	265.00	269.00	266.00	Durvalumab + EP	SCLC	13	10.3	5.1	5.4
KEYNOTE-604 study	453	228.00	223.00	225.00	223.00	Pembrolizumab + EP	SCLC	10.8	9.7	4.5	4.3
Total	2671.00	1263.00	1248.00	1262.00	1246.00		Mean	11.775	10.3	4.85	4.6

## Data Availability

Phase III randomized controlled trials compared ICI plus etoposide and platinum versus etoposide and platinum, as the first-line SCLC. They were considered eligible. The authors searched for English published studies on PubMed with the keywords Nivolumab, Ipilimumab, Atezolizumab, Pembrolizumab, Checkmate, impower, small-cell lung cancer, keynote, and lung cancer. The authors also searched on Google Scholar for the latest update for oncology conferences, such as the American Society of Clinical Oncology (ASCO). They used the latest and the most completed data in case of duplication, and the last search date was 07/15/2020.

## Supplementary Materials

Supplementary Materials The supplementary files contain forest plot figures that do not exist in the manuscript and forest plot of subgroups. They contain the table that has all the details of included studies in addition to the flow diagram and risk of bias assessment. Each figure and table in the supplementary file has reference to it in the manuscript.
