# Supplementary material for: Combination of the EP and Anti-PD-1 Pathway or Anti-CTLA-4 for the Phase III Trial of Small-Cell Lung Cancer: A Meta-Analysis
Source: J Oncol. 2021 May 22;2021:6662344. doi: 10.1155/2021/6662344 (PMC8166470; doi:10.1155/2021/6662344)
Supplement: Supplementary Materials — The supplementary files contain forest plot figures that do not exist in the manuscript and forest plot of subgroups. They contain the table that has all the details of included studies in addition to the flow diagram and risk of bias assessment. Each figure and table in the supplementary file has reference to it in the manuscript. [file 6662344.f1.docx]

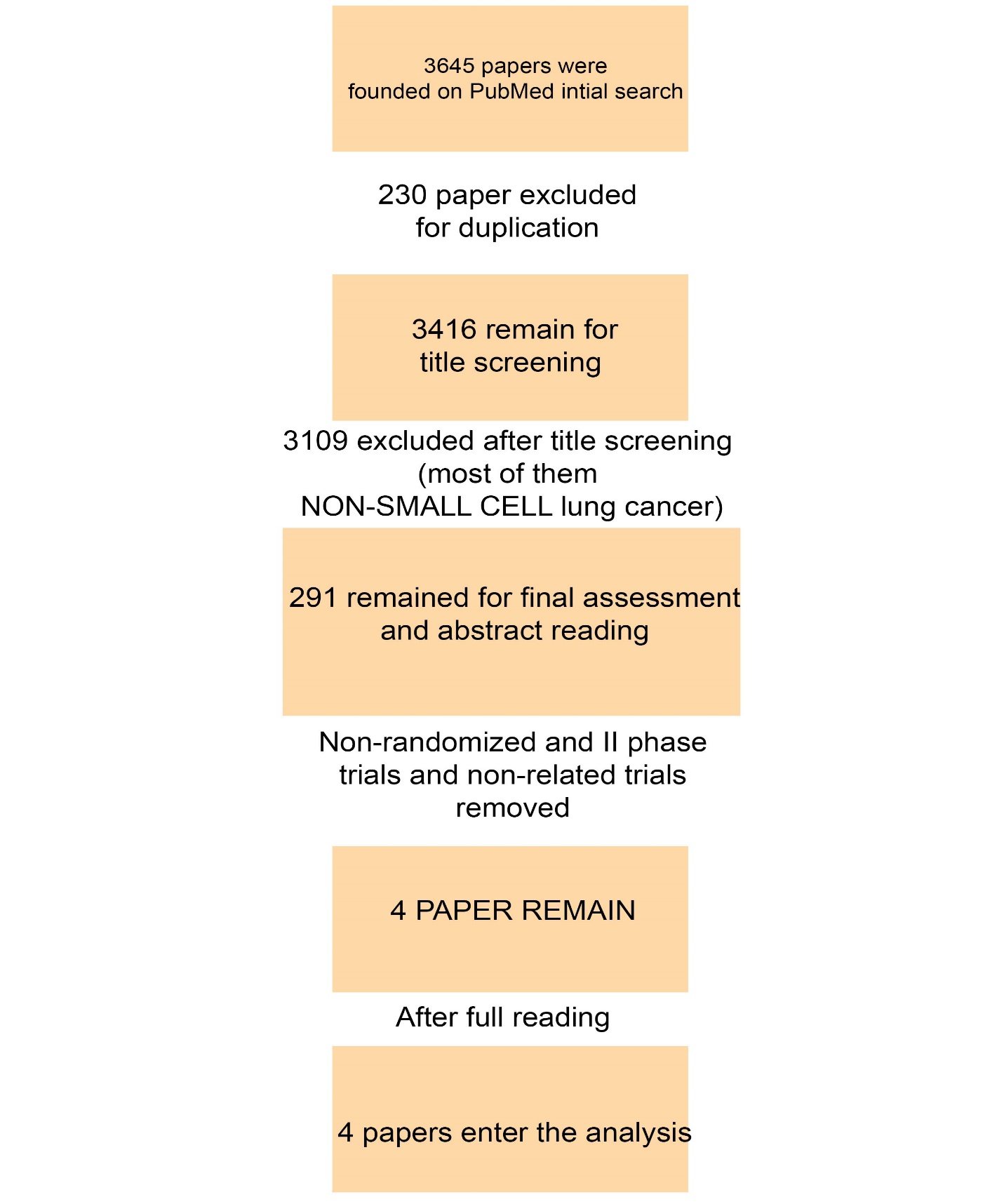


Figure 1 :Flow diagram showing the trial selection process for the systematic review and meta-analysis.

Figure 2: risk of bias result by using ROB2.0 TOOL.


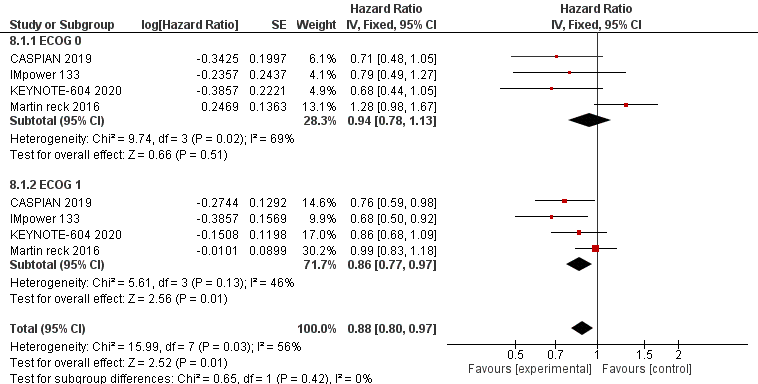


Figure 3: overall survival rate of subgroups according ECOG VALUE.


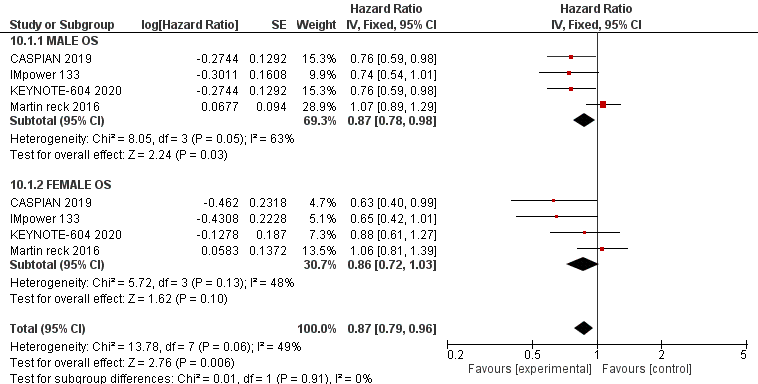


Figure 4: overall survival rate of subgroups according to gender difference.


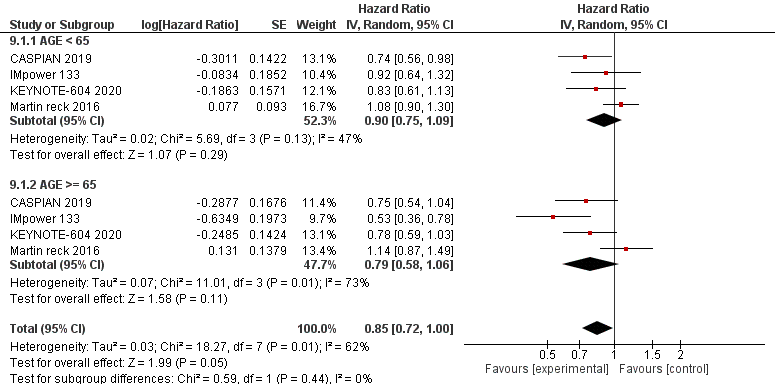


Figure 5: overall survival rate of subgroups according to age differences.


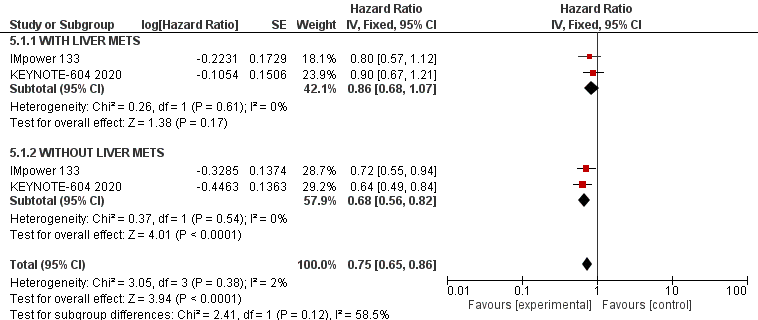


Figure 6: progression free survival rate hazard ratio with and without liver metastasis.


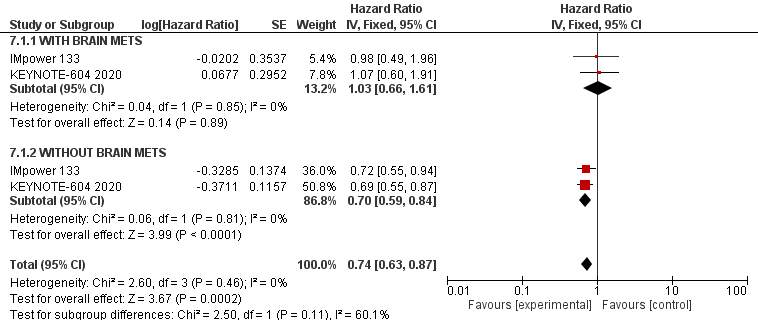


Figure 7: progression free survival hazard ratio with and without brain metastasis.


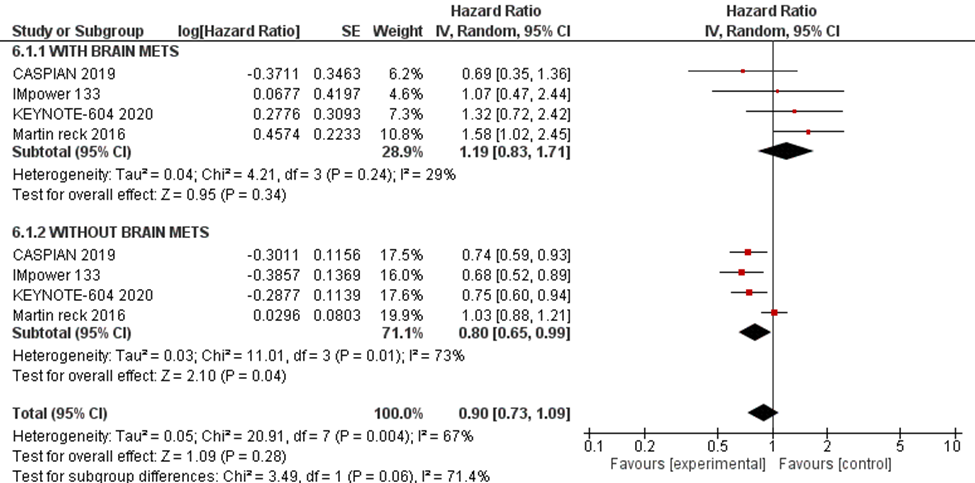


Figure 8: the overall survival rate different between with and without brain metastasis.


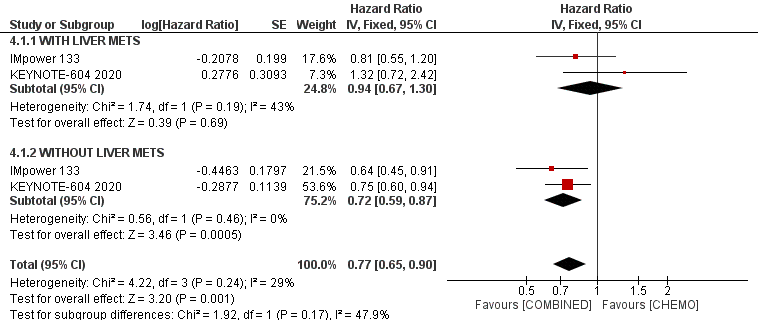


Figure 9: Overall survival rat different between with and without liver metastasis.

Table 1: full patient and studies characteristics of all included studies.

|  | Median age |  | <65 yr |  | >=65 |  | Male |  | female |  | ECOG0 |  | ECOG1 |  | Never smoked |  | Current smoker |  | Former smoker |  | Brain metastasis |  | Liver metastasis |  | Complete response |  |
| --- | --- | --- | --- | --- | --- | --- | --- | --- | --- | --- | --- | --- | --- | --- | --- | --- | --- | --- | --- | --- | --- | --- | --- | --- | --- | --- |
|  | comb | EP | comb | EP | comb | EP | comb | EP | comb | EP | comb | EP | comb | EP | comb | EP | comb | EP | comb | EP | comb | EP | comb | EP | comb | EP |
| KEYNOTE-604 Study | 64 | 65 |  |  | 113 | 124 | 152 | 142 |  |  | 60 | 56 | 168 | 169 | 8 | 8 | 148 | 133 | 72 | 84 | 33 | 22 |  |  | 4 | 2 |
| CASPIAN 2019 small cell | 62 | 63 | 167 | 157 | 101 | 112 | 190 | 184 | 78 | 85 | 99 | 90 | 169 | 179 | 22 | 15 | 120 | 126 | 126 | 128 | 28 | 27 | 108 | 104 | 6 | 2 |
| IMpower 133 2018 small cell | 64 | 64 | 111 | 106 | 90 | 96 | 129 | 132 |  |  | 73 | 67 | 128 | 135 | 9 | 3 | 74 | 75 | 118 | 124 | 17 | 18 |  |  | 5 | 2 |
| Martin reck 2016 ipi + etoposid and platinum small cell | 62 | 63 | 299 | 277 | 179 | 199 | 317 | 326 | 161 | 150 | 137 | 147 | 340 | 328 |  |  |  |  |  |  |  |  |  |  | 1 | 0 |

| a | Median age |  | <65 yr |  | >=65 |  | Male |  | fremale |  | ECOG0 |  | ECOG1 |  | Never smoked |  | Current smoker |  | Former smoker |  | Brain metastasis |  | Liver metastasis |  | Complete response |  |
| --- | --- | --- | --- | --- | --- | --- | --- | --- | --- | --- | --- | --- | --- | --- | --- | --- | --- | --- | --- | --- | --- | --- | --- | --- | --- | --- |
|  | comb | EP | comb | EP | comb | EP | comb | EP | comb | EP | comb | EP | comb | EP | comb | EP | comb | EP | comb | EP | comb | EP | comb | EP | comb | EP |
| IMpower 133 2018 small cell | 64 | 64 | 111 | 106 | 90 | 96 | 129 | 132 |  |  | 73 | 67 | 128 | 135 | 9 | 3 | 74 | 75 | 118 | 124 | 17 | 18 |  |  | 5 | 2 |
| KEYNOTE-604 Study | 64 | 65 |  |  | 113 | 124 | 152 | 142 |  |  | 60 | 56 | 168 | 169 | 8 | 8 | 148 | 133 | 72 | 84 | 33 | 22 |  |  | 4 | 2 |
| CASPIAN 2019 small cell | 62 | 63 | 167 | 157 | 101 | 112 | 190 | 184 | 78 | 85 | 99 | 90 | 169 | 179 | 22 | 15 | 120 | 126 | 126 | 128 | 28 | 27 | 108 | 104 | 6 | 2 |
| Martin reck 2016 ipi + etoposid and platinum small cell | 62 | 63 | 299 | 277 | 179 | 199 | 317 | 326 | 161 | 150 | 137 | 147 | 340 | 328 |  |  |  |  |  |  |  |  |  |  | 1 | 0 |
